# Influence of alphaxalone on motor somatosensory evoked potentials in a female rhesus macaque (*Macaca mulatta*)

**DOI:** 10.1177/0023677221990706

**Published:** 2021-02-09

**Authors:** Henri Georges Michel Justin Bertrand, Joseph Adam Middleton, Stuart Nicolas Baker, Isabel Glover, Paul Andrew Flecknell

**Affiliations:** 1 University Biomedical Services, University of Cambridge, UK; 2 Faculty of Life Science and Medicine, King’s College London, UK; 3 Institute of Neuroscience, Newcastle University, UK

**Keywords:** Alphaxalone, somatosensory evoked potential, neurosurgery, primate

## Abstract

This communication reports the effect of alphaxalone on motor somatosensory evoked potential (SEPs) in a rhesus macaque. The animal was deeply anaesthetised with an infusion of ketamine, medetomidine, midazolam and alfentanil. The median nerve was stimulated, and SEPs were recorded from the motor cortex. The successive administration of three doses of alphaxalone (0.5, 1 and 2 mg/kg) induced an increase of the latency time and a decrease of the amplitude of the SEPs. However, the structure of the waveforms was conserved, and hence alphaxalone might represent a suitable general anaesthetic option in neuroscience research as well as veterinary or human medicine.

## Résumé

Cette communication rapporte l’effet de l’alfaxalone sur le potentiel évoqué moteur somesthésique (PES) du macaque rhésus. L’animal a été profondément anesthésié par une perfusion de kétamine, de médetomidine, de midazolam et d’alfentanil. Le nerf médian a été stimulé et les PES ont été enregistrés à partir du cortex moteur. L’administration successive de trois doses d’alphaxalone (0.5, 1 et 2 mg/kg) a induit une augmentation du temps de latence et une diminution de l’amplitude des PES. Toutefois, la structure des formes d’onde a été conservée et, par conséquent, l’alphaxalone pourrait représenter une option anesthésique générale appropriée pour la recherche en neurosciences ainsi que la médecine vétérinaire ou humaine.

## Abstract (de)

Diese Mitteilung informiert über die Wirkung von Alfaxalon auf motorisch-somatosensorisch evozierte Potenziale (SEP) bei einem Rhesusaffen. Das Tier wurde mit einer Infusion mit Ketamin, Medetomidin, Midazolam und Alfentanil tief narkotisiert. Der Nervus medianus wurde stimuliert und SEP aus dem motorischen Kortex aufgezeichnet. Die sukzessive Verabreichung von drei Dosen Alfaxalon (0,5, 1 und 2 mg/kg) induzierte eine Verlängerung der Latenzzeit und eine Abnahme der Amplitude der SEP. Die Struktur der Wellenformen blieb jedoch erhalten, so dass Alfaxalon eine geeignete Option für die Allgemeinanästhesie in der neurowissenschaftlichen Forschung sowie in der Veterinär- oder Humanmedizin darstellen könnte.

## Resumen

Este informe refleja el efecto de alfaxalona sobre potenciales evocados somatosensitivos (SEP) motores en macacos Rhesus. El animal fue anestesiado mediante una infusión de ketamina, medetomidina, midazolam y alfentanil. El nervio mediano fue estimulado y se registraron SEP de la corteza motora. La administración sucesiva de tres dosis de alfaxolona (0,5, 1 y 2 mg.kg^−1^) indujo un aumento del tiempo de latencia y una disminución de la amplitud de los SEP. Sin embargo, la estructura de la formación de ondas fue conservada y, por tanto, la alfaxalona puede representar una opción de anestesia general adecuada en la investigación de neurociencia así como en la medicina veterinaria o humana.

## Introduction

The principal form of communication between neurons is the action potential generated by ion transport across the neurone cell membrane. Somatosensory evoked potentials (SEPs) are action potentials produced when one of the peripheral sensory receptors or an afferent nerve of the somatosensory system (e.g. touch, pain, kinaesthesia) is stimulated over their resting threshold. SEPs can be recorded at the level of the contralateral somatosensory cortex and are an indicator of the integrity of the various components of the afferent somatosensory pathway.^1,2^ SEPs are widely used in the clinical setting during spinal surgery, such as the correction of scoliosis, as well as in research for pain and neuroplasticity studies.^1–3^ Anaesthetics can affect amplitude and latency in a dose-dependent manner, particularly halogenated agents, which produce the most interference with SEPs.^4^ As a result, injectable agents such as ketamine and α_2_-agonists are preferred.^3–5^ Alphaxalone (3α-hydroxy-5α-pregnane-11,20-dione) is a short-acting neurosteroid anaesthetic with no cumulative effect.^6^ This communication reports the effect of alphaxalone on motor SEPs in a rhesus macaque.

## Methods

### Ethical statement

The use of animals for research was authorised by the UK Home Office (PPL60/4560) and by the Newcastle University Animal Welfare and Ethical Review Body.

### Animal

One adult female rhesus macaque (four years old, body weight 7 kg) was involved in this study. The animal was pair-housed in indoor pens with a solid floor (minimum of 4.40 m^2^) and windows, allowing a view of the other pens and the corridors. Enrichment devices and substrate for foraging were provided.

### Anaesthesia procedure

To conduct motor SEPs recordings, the primate was sedated with 10 mg/kg intramuscular ketamine (Narketan 10, 100 mg/mL; Vetoquinol UK Ltd, Towcester, UK), and anaesthesia was induced with slow intravenous administration of 6 mg/kg propofol (Fresenius Propoven, 1%; Fresenius Kabi Ltd, Runcorn, UK) to allow endotracheal intubation. Anaesthesia was maintained with intravenous infusion of 6–8.6 mg/kg/h ketamine, 0.2–0.6 μg/kg/min alfentanil (Alfentanil, 500 μg/mL solution for injection; Hameln Pharmaceuticals, Gloucester, UK) and 1–3.56 μg/kg/h of medetomidine (Domitor® 1 mg/mL solution for injection; Vetoquinol UK Ltd). The animal was connected to a circle breathing system (Clear-Flo™; Intersurgical Ltd, Wokingham, UK) and the lungs were mechanically ventilated (Merlin Small Animal Ventilator; Vetronic Services Ltd, Abbotskerswell, UK). Physiological parameters (electrocardiogram, SpO_2_, invasive blood pressure, rectal temperature, EtCO_2_, gas analyser) were constantly monitored with a Vitalogik 4500 monitoring system (Charter-Kontron Ltd, Milton Keynes, UK).

### SEPs recording protocol

While the animal was anaesthetised, two external electrodes (3M™ Red Dot™ Repositionable Monitoring Electrode 2660-3) were placed over the median nerve route to stimulate it. The intensity of stimulation was equivalent to two-and-a-half times the motor threshold. This level of stimulation is usually optimal to activate all group I and II afferents without causing pain. An epidural recording of the SEPs resulting from median nerve stimulation was performed by the apposition of a dipolar ball electrode on the dura of the primary motor cortex (M1) and somatosensory cortex (S1) regions (gain 50 K, bandpass 0.5 Hz–2 KHz, sampling rate 5 KHz). Stimulus markers and SEPs were sampled using a micro1401 interface and Spike2 software (Cambridge Electronic Design, Cambridge, UK).

### Alphaxalone administration

After baseline waveform recording for three minutes, three intravenous boluses of alphaxalone (Alfaxan, 10 mg/ml solution for injection for dogs and cats; Jurox UK Ltd, Crawley, UK) at 0.5, 1 and 2 mg/kg were successively administered. After the administration of each incremental dose, SEPs were recorded for a period of 1000 seconds, and a washout period of five minutes was allowed for the waveform parameters to return to baseline.

### Waveforms analysis

From the recorded SEP waveforms, two parameters were analysed: the latency representing the time from the stimulation to the first peak and the amplitude of this peak. The Friedman test was used to compare the P1 amplitudes and latencies between the three alphaxalone doses. Statistical analysis was performed with GraphPad version 7.0d (GraphPad Software LLC, La Jolla, CA). A *p*-value of <0.05 was considered statistically significant.

## Results

**Figure 1.**
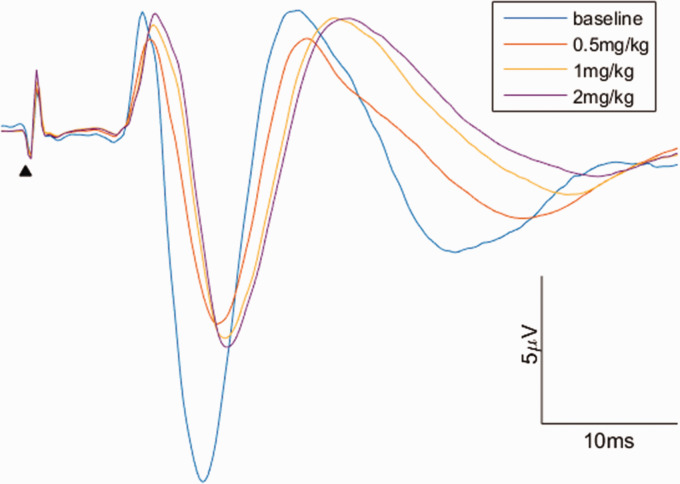
Influence of alphaxalone on motor SEPs waveform. The waveforms for the figure were obtained by merging all the individual waveforms recorded for each alphaxalone concentration. The arrow (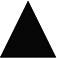) highlights the peak corresponding to the stimuli. The latency and amplitude were, respectively: at baseline, 8.6±0 ms and 16.3±0.5 μV; at 0.5 mg/kg, 9.1±0.1 ms and 10.1±0.7 μV; at 1 mg/kg, 9.4±0.1 ms and 11.1±0.5 μV; at 2 mg/kg, 9.5±0.1 ms and 11.5±0.4 μV.

The intravenous administration of each bolus of alphaxalone did not modify the primate’s heart rate and blood pressure. However, after the administration of each bolus, a significant modification of P1 amplitude (*p*<0.0001) and latency (*p*<0.0001) compared to baseline was observed (Figure 1).

## Discussion

This report shows that alphaxalone influences the amplitude and latency of motor SEPs, but the recorded waveform was conserved and can be easily analysed at doses at least up to 2 mg/kg. Interestingly, the amplitude of the SEPs recorded increased with successive doses, and hence habituation of the central nervous system to the effects of alphaxalone cannot be ruled out. Also, the other components of the balanced anaesthetic regimen administered when recording baseline responses may have influenced the SEPs. The use of ketamine, midazolam and opioids has been described and used to record motor SEPs in rhesus macaque.^7^ Alphaxalone is a general anaesthetic acting at the GABAa receptor, resulting in the hyperpolarisation of the neuron and inhibition of action potentials.^8^ This anaesthetic was widely investigated in dogs and cats, and it has wide safety margins in these species with hypoventilation and apnoea as the main complications.^9,10^ The use of alphaxalone in combination with other anaesthetics to immobilise macaques has also been described, where the highest dose of 2 mg/kg administered in this report was similar to those previously reported.^11,12^ Currently, alphaxalone is only available for veterinary use, but a formulation was previously available for human anaesthesia known as Althesin. The use of Althesin was considered suitable for human neuroanaesthesia.^13,14^ A new formulation of alphaxalone is currently entering Phase III clinical trials in humans (https://adisinsight.springer.com/trials/700292315).

In conclusion, despite the alteration of motor SEPs parameters, the use of alphaxalone may be a useful agent in neuroscience research and could represent an alternative to ketamine which is becoming subject to greater access control worldwide.^15^ However, further work is required to establish an optimal anaesthesia regimen, dependent on the medical or scientific objectives.
